# Reflections on the concept of subject

**DOI:** 10.3389/fsoc.2026.1874290

**Published:** 2026-10-14

**Authors:** Michela Luzi

**Affiliations:** Università Niccolò Cusano, Rome, Italy

**Keywords:** human rights, individualism, social movements, subject, triangle of differences

## Abstract

In contemporary society, new social movements with a cultural focus have emerged, addressing themes such as sexuality, information, ecology, and gender equality. These movements represent a new form of protest, distinct from the revolutionary and conflict-driven tendencies of traditional movements. For these new social movements to become effective agents of societal transformation, collaboration is essential, along with the support of the “Soggetto,” which represents individuals upheld by rights and institutions. Advocating human rights can be a powerful strategy if it serves as a framework to restore meaning in society, fighting the persistent socio-cultural drift that enforces standardized and homogenized roles. This approach, coupled with an understanding of complex interdependence, can help mitigate the effects of disintegrative imperialism and address social crises.

## Introduction

The civilization of work and organizations, which reached its zenith in the modern era, now finds itself on the brink of a new revolution. This revolution is defined by a conflict between the capitalist system of exchange and the role of meanings (Piketty, 2019). A rift has emerged between the instrumental approach, as defined by Weberian rationality, and the system of identities. This division has led to an erosion of values and perspectives, underscoring the necessity to reconstruct society by reconciling the dichotomy between financial and economic instrumentalism and cultural, social, and political identifications.

Decades of globalization, driven primarily by economic criteria, have fostered complex interdependence, facilitating the rise of disintegrative individualism. This individualism has increasingly separated instrumental reasoning from identity formation (Held and McGrew, 2000; Gallino, 2003; Bixio, 2013). The process of de-modernization has further fractured identities, encompassing dimensions such as being, memory, tradition, language, gender, age, economic activities, and technical skills (Bauman, 2001). The extreme consequence of this trend is desocialization, marked by the disappearance of roles, norms, and social values that shape our lived experiences (Touraine, 2006). As desocialization progresses, society—once regulated by institutions and their roles—now finds itself guided by market forces and the initiatives of economic actors who dominate the landscape (Morin, 1985). This fracture between the economy and the broader social fabric emphasizes the crucial role of human experience. Despite being constrained by economic necessity, individuals can strive to transcend this dichotomy by setting goals and forming movements that challenge prevailing trends (Ricciardi, 2011). This need for action is especially urgent when faith in unconditional progress has waned, prompting a search for new paradigms to address the entropies within our economic, social, and political systems.

### The sociology of action and the new movements

French sociologist Alain Touraine, in two significant works, *Sociologie de l’action* (1965) and *La conscience ouvrière* (Touraine, 1960; Touraine, 1966), addresses an interpretive need that contributed to the emergence of the “sociology of action.” This field distinguishes itself from sociology of values by focusing on the role and commitment of social actors, referred to as the “Soggetto.” Touraine interprets worker consciousness as a phenomenon where individuals define and recognize themselves as workers, aware that they embody three fundamental principles:

1. Identity Principle: Workers see themselves as producers and contributors of an essential factor in production.
2. Opposition Principle: They identify adversaries, recognizing obstacles that prevent them from reaping the rewards of their labor.
3. Totality Principle: This principle encompasses the realm of social conflicts (Oommenn, 1996).

This analysis of working-class consciousness reveals two constants that challenge the notion of a homogeneous working class, typically portrayed as victims of capitalist oppression (Harvey, 2014, 2021). On one hand, workers should not be viewed as a monolithic group; on the other, they struggle for autonomy against the domination of labor and employers, leading to movements that address not only workplace issues but also broader social and political demands (Touraine and Wieviorka, 1986; Gombin, 1970; Johnston, 2014). The sociology of action has contributed to the formation of social movements that emerged from the upheavals of the 1960s (Waters, 2008). For example, university movements transcended campus boundaries, gaining international momentum and leading to the identification of the underlying causes of their struggles. These movements became the precursors of new social problems that have gained significance since the 1970s (Melucci, 1975, 1985). University movements arose for specific social reasons: students from mass universities, often from petite bourgeois backgrounds, entered adulthood not as “privileged youth” but as individuals aware of their socio-economic disadvantages. They sought social mobility through education, only to discover that many courses did not guarantee stable professional opportunities. Lacking the necessary social capital for integration into the ruling class, these students recognized that their employment prospects depended on broader social structures, which prompted the formation of movements.

These struggles quickly expanded beyond the university context, as activists sought to engage other traditionally marginalized groups dissatisfied with their circumstances (Antonelli, 2009). The emergence of this new form of resistance, characterized by persistent activism across various countries, led to claims that resonated beyond academic institutions and contributed to social and political crises (Morin et al., 1968; McCarthy and Zald, 1973, 1977). Alongside student movements, other significant movements—such as regionalist, feminist, and anti-nuclear movements—also gained prominence, organized through collective action that transcended national borders and engaged with international issues (Olson, 1965; Baldassarri, 2009).

The 1960s and 1970s were marked by substantial changes, reforms, collective protests, cultural shifts, and radical transformations, making sociological observation essential (Melucci, 1978, 1980). Collective action, whether emerging from individual decisions or organizational structures, often centers on political decision-making (Smelser, 1962; Sovacool, 2022). Consequently, social movements aiming to develop a collective identity that is represented in political discourse become crucial (Polletta and Jasper, 2001). A social movement is generally defined as an informal network of individuals and groups who share common beliefs, trust each other, and collectively engage in conflict through frequent protests (de Nardis, 2023). This specificity makes social movements valuable, as they reveal a unique form of collective action that enables a social category to challenge any form of domination, while appealing to broader societal values (Touraine, 1981, 2007).

Social movements seek to “re-naturalize” society by opposing the hegemony of traditional decision-making centers and advocating change as a planned, controllable process (Cohen, 1985; Blings, 2020). However, not all social mobilizations, protests, and collective actions meet the criteria of social movements. True social movements thrive in democratic societies, existing when political institutions maintain a degree of autonomy. Under these conditions, movements represent collective behaviors that challenge society’s use of cultural resources, knowledge models, production forms, and moral principles (Ayhan and Bee, 2024).

According to Alain Touraine, a social movement is defined as “la conduite collective organisée d’un acteur de classe luttant contre son adversaire de classe pour la direction sociale de l’historicité dans une collectivité concrète” (Touraine, 1973: p. 368). This definition suggests that a movement encompasses three elements akin to the principles of working-class consciousness: the defense of the movement’s identity and interests (Identity Principle), the realization of this awareness through conflictual relationships with an adversary (Opposition Principle), and a shared vision that unites participants (Totality Principle). Consequently, a social movement cannot exist without a defined counter-movement (Goodwin et al., 2001).

The traditional conflict stemming from working-class consciousness has gradually evolved into a phase of disintegration, resulting in a crisis of the classic labor movement, which can no longer symbolize social conflict as it did in industrial society (Ding and Hirvilammi, 2024). This crisis reflects a broader configuration of social life, marked by some social movements becoming weaker while others have turned to terrorism or early institutionalization, transforming into pressure groups (Castells, 2012; Pirzio Ammassari and Marchetti, 2018). Such developments have led to a decline in social movements, as they have slowed their initiatives to achieve concrete results. Unlike the original labor movement, characterized by focused, militant groups, new social movements are shaped within the democratic processes of public opinion formation and are less active in political confrontation (Habermas, 1981; Johnston, 2014; Hutter et al., 2019; Cammaerts, 2021).

These new movements are no longer tethered to issues of work, income, or wages; instead, they are based on diverse cultural foundations and expectations, addressing topics such as sexuality, information, religion, nuclear energy, the environment, and gender (Eder, 1985; Melucci, 1989; Sovacool, 2022). This shift distances them from the revolutionary intentions of the movements that characterized the 1960s (Castells, 2012). By the end of the 1960s and into the 1970s, the working-class movement was nearing its conclusion as a social movement, even though Crouch and Pizzorno (1978) identified a revival of class conflict during that period. “At the same time, new forms of protest were coming to the fore, to the point that it was quite legitimate to postulate a change in the type of society. From this point of view, there was a move from the industrial to post-industrial era, and the protest movements in post-industrial societies were no longer the working-class movement, historically on the wane, but the struggles of students, anti-nuclear groups, regionalist groups, women, and so on” (Wieviorka, 2005a, p. 5).

Participation in movements can arise from various motivations, both individual and socio-structural. Not everyone joins out of moral beliefs; many participate due to connections with friends or acquaintances already involved, or for professional interests (Daher, 2013). Others seek a sense of identity and belonging (Gongaware, 2010). The new social movements have emerged as platforms for individuals who, disenchanted with traditional political participation, seek alternative forms of expression and identification (McDonald, 2002; Della Porta et al., 2017). This shift has enabled new movements to establish themselves even amid the erosion of common sentiment, propelled by individualization and cultural transformations, thereby becoming vital forms of identity expression (Millefiorini, 2002). The challenge of finding stable institutions that can adequately respond to individuals’ identity needs has contributed to an increasingly fragmented and uncertain contemporary society (Chouhy, 2020). This fragmentation has fostered an emphasis on individual self-affirmation while weakening social bonds and responsibilities, leading to disaffection with traditional, political, and social participation (Alteri et al., 2016).

Modernization has fundamentally reshaped the subjectivity of movement participants. Historical analyses and empirical studies reveal that the German labor movement was characterized by a reliable collective subjectivity, while new social movements emerged from a more mobile yet solid individual subjectivity (Lösche and Walter, 1989). In contrast, today’s small-scale, local, and experimental movements reflect a flexible and fractured subjectivity (Blühdorn et al., 2018). This ‘liquefaction’ of the subject (Bauman, 2012) “signals two developments: (a) direct social actions might no longer predominantly serve to prepare an alternative social order but to perform and experience it within the existing one; (b) with the subjective impetus of direct social action altered in that way, teaming up with a political party that could help implementing a social utopia is now longer deemed necessary” (Butzlaff and Deflorian, 2021, p. 4).

The transformation in the structure and dynamics of movements highlights the need for a sociology of action. In contemporary societies, the dichotomous opposition between rationalizers and consumers, or capitalists and workers, is diminishing. Everyone strives to improve their personal situations and secure adequate access to the consumer market. This historical opposition is no longer functional to prevailing economic conditions and hinders the emergence of new social movements, as claims now focus more on sharing benefits than on societal orientations (Rossi, 2018; Cammaerts, 2021).

Social movements extend beyond mere conflicts of interest; they also pertain to resources and values, gaining strength only when closely associated with the subject (Touraine, 2010). Thus, the centrality of the Subject—understood as action and relation—is crucial in the emergence of new social movements and their distinctive forms of conflict (Cousin and Rui, 2011). Social actors and movements that once protested against traditions, conventions, and elitist privileges now direct their efforts against apparatuses, discourses, and uncertainties related to environmental sustainability. These challenges prevent the realization of projects and the establishment of specific objectives, shifting the focus of engagement in conflicts, debates, and negotiations (Ricci, 2024). As a result, the arena of confrontation and the scope of issues have evolved, calling traditional roles and functions into question.

### Some possible functions of the subject

Starting with the analysis of new types of social movements closely linked to individual needs and desires, we recognize the significant role the Subject plays in society, increasingly viewed as a paradigm by sociologists (Lojkine, 2015; Blühdorn, 2021). Because, “completamente proteso verso la propria capacità di agire, la propria creatività – esso è artefice della propria esistenza, domina la propria esperienza, dispone (per utilizzare un termine dell’economista Amartya Sen) di *capabilities*, di risorse per agire scegliendo il proprio modo di vivere (Sen, 1987; Naz, 2020). Questa idea di soggetto si distingue dall’individualismo contemporaneo come da ogni forma di utilitarismo per il fatto che tiene conto di come, se io posso essere soggetto, bisogna che possano esserlo anche tutti gli altri esseri umani – una riflessione che getta un ponte verso la riflessione sulla convivenza sociale e sulla democrazia” (Wieviorka, 2021: p. 210).

According to this definition, the individual, as Subject, can identify with social norms, institutions, and their community, allowing them to challenge social dynamics in contexts where less recognizable and more insidious forms of domination can lead to de-subjectivation (Hansell and Grassie, 2011; Dubet and Wieviorka, 2018). Constructing an identity based on belonging has become increasingly complex, as communities often appear static and unable to fulfill the desire of identification (Crespi, 2015; Kinna, 2019; Yilmaz and Bağçe, 2022).

The challenges of building one’s identity lead to an erosion of certainties, creating confusion due to the individual’s feeling of being a passive actor within their community (Mc Garry and Jasper, 2015; Bauman, 2020). This can result in marginalization and alienation, where individuals must choose between passively accepting societal offerings or finding the moral strength to emancipate themselves from mechanistic conditions, combating a pervasive sense of emptiness (Butler, 2005). To pursue the latter, it is crucial to break free from market constraints and reimagine communal living. The prevailing creed of economist—often viewed as the unacknowledged ruler of our times—has revealed its limitations, relegating individuals to discomfort (Simmel, 2004: Lojkine, 2009; Scott, 2013). The promises of consumer culture have proven to be illusions, overshadowed by the fluidity of contemporary relationships and practices.

“Ce n’est plus le Sujet triomphant de la Déclaration des droits de l’homme que nous rencontrons aujourh’hui dans notre univers déchiré par des guerres devenues civiles, c’est un Sujet luttant pour sa survie, seule force de résistance à la rupture complète et définitive entre le monde des marchés et celui des communautés. Force fragile, à la merci à la fois de la culture de masse et de l’autoritarisme communautaire, et pourtant force à partir de laquelle, partout dans le monde, s’amorcent des tentatives de construction de la vie personnelle et de la vie sociale” (Touraine, 1997: p. 96).

This dynamic position the Subject as a dual actor, engaging in both production and culture—the two central spheres of social reality that facilitate personal development through balanced adaptation and assimilation (McDonald, 1994; King, 2006). This is especially pertinent in contemporary societies characterized by significant conflict, where individuals must navigate the dualities of economic interests and cultural identities (Touraine, 2002; Streeck, 2016; Brayne, 2020).

The predominance of economic interests, intended to liberate humanity from need, has led instead to the fragmentation of social dimensions, favoring a cynical individualism that no longer fosters growth or well-being (Zuboff, 2019; Gallino, 2003). This situation distances individuals from their humanity, undermining the relational depth necessary for social actors, rendering the current crisis distinct from past ones (Bowman and Pickard, 2021).

Today’s crisis requires a dual transformation: of society, which remains in perpetual crisis (Touraine, 2013; Burrell and Fourcade, 2021), and of the Subject, who, feeling weakened and lacking a cohesive identity, must identify a new paradigm for proper social placement (Wieviorka, 2008; Hitachi-UTokio Laboratory, 2020). In seeking this paradigm, individuals can rediscover and value their subjectivity through the respect and defense of human rights, shaping new dimensions of relationships among diverse individuals.

Overcoming the current crisis is challenging without genuinely defending the universal rights of the Subject, while also safeguarding the diverse identities within a globalized society (Appadurai, 1996; Diamond, 2024; Draçi and Kadiu, 2024). Such rights can thrive only in societies where democracy is recognized as an irreplaceable universal good (Marchetti, 2018). In democratic contexts, human and emerging rights find their natural home, allowing individuals to act as conscious social subjects. Democracy today seems to lack meaning and can regain it only through the cultivation of democratic actors; without convictions, institutions alone cannot function (Crouch, 2005; Clemens, 2016).

Democracy, despite its increased complexity, remains essential as it uniquely promotes individual freedom, enabling the affirmation of equality and justice (Wenman, 2013; Acemoglu et al., 2019; Ragazzoni, 2022). The urgency of centering the Subject arises from the need for human conduct driven by conscience rather than mere conformity (Urbinati, 2011; Milanovic, 2016). These actions connect to the affirmation of individuals striving to overcome obstacles and evade new forms of obstructionism that hinder their pursuit of freedom and the protection of human rights (Yilmaz and Bağçe, 2022).

Recognizing universal human rights is thus a crucial condition, serving as a bulwark against any attempt at oppression (Nussbaum, 1997, 2011). The individual’s focus on their ability to act, coupled with creativity, positions them as the architect of their existence, equipped with resources conducive to their chosen way of living—steering clear of the individualism that pervades contemporary social reality (Beck and Beck-Gemsheim, 2001; Sánchez, 2010). This individualism manifests as a demand for participation in consumption, employment, education, healthcare, and wealth—elements viewed as prerequisites for acceptance. Unfortunately, the long-term consequences of this isolating trend have not been adequately considered, resulting in the disintegration of collective life and community, and fostering dramatic phenomena like racism, ongoing conflict, inequality, and even war (Pyythinen, 2008; Blühdorn et al., 2018).

This *scenario* prompts reflection on how the ideal Subject may conflict with other actors who assert their subjectivity while denying that of others. Certain social actors adopt attitudes that essentialize their groups, compromising relationships and enabling logic rooted in indifference and distance, which may escalate into aggressive and violent behaviors characteristic of the negative aspects of the Subject: the anti-subject (Wieviorka, 2015). “The *Anti-*Subject is that side of the Subject that fails to acknowledge the other person’s right to be a Subject and which can develop only by negating the other person’s humanity. This case corresponds to the dimensions of cruelty or enjoyment of violence for its own sake, as an end in itself. Here, the victim is dehumanized, reified, or animalized and is in every respect the opposite of the Subject” (Wieviorka, 2012b, p. 20).

The behaviors of the non-subject or anti-subject lead to destruction, the denial of others, and violence, often accompanied by dehumanization and objectification. A comprehensive analysis of the Subject must consider both its positive and negative dimensions, along with the potential risks and responsibilities entailed (Zaru, 2020). Each Subject is shaped by life experiences, education, and choices, including those not made. “Ces processus sont de deux ordres, qu’il faut distinguer analytiquement même s’ils peuvent se combiner contradictoirement dans la pratique: les uns sont des processus de subjectivation, les autres de dé-subjectivation. […] La subjectivation conduit vers le «sujet» à la Touraine ou à la Joas, capable d’agir car capable de se penser comme acteur et de trouver les modalités du passage à l’action, la désubjectivation conduit à l’inverse vers les formes décomposées et inversées su sujet, vers l’anti-sujet ou le non-sujet, et, de là, éventuellement, vers des conduites de destruction et d’autodestruction” (Wieviorka, 2012a, p. 6).

Often, the anti-subject and de-subjectivation foster phenomena like hatred, racism, terrorism, and anti-Semitism, which reject universalism, humanism, and creativity in favor of destruction and self-destruction (Geys and Qari, 2017). This struggle takes place in the daily lives of disoriented and vulnerable individuals within fragmented societies that have eroded the sense of community (Bauman, 2020).

### Functions of the difference triangle

The recent and sudden social changes, which have generated uncertain and contradictory situations, underscore the urgency of establishing a democratic culture grounded in the defense of individual rights and the protection of social actors against various forms of power (Wenman, 2013; Marchetti, 2018; Schneider and Thomson, 2024). The defense of human and social rights must become a priority, both individually and collectively, as a means to restore meaning to society (Milanovic, 2016). This approach can help counteract the socio-cultural drift that imposes standardized roles on individuals, hindering the full realization of their subjectivity (Touraine, 2015). “My essence as a subject (not as an organic being, of course, but as an actor, as a subject of opinions and rights, as a bearer of emotions and relations) is founded on my ability to exercise my multifaceted capacities by relating to the world. The consistency of my own person is the consistency of my world. My own subjective coherence is grounded in the coherence of my relations and worldviews. My contradictions work themselves out in the tensions and divisions of my world. This process must not be understood as a flat projection or reflection of the ego onto the world, nor as an abstract metaphysical idealism. It is rather a creative and open process in which we become ourselves only by shaping our environment. Our being consists in the complex unfolding of personal autonomy through perception, exploration, and action” (Pugliese, 2021: p. 76).

Starting from the biographical traits of the Subject’s identity, we can initiate collective action, allowing members of groups with common interests and mutual solidarity to define themselves and give meaning to their unified efforts.

In this sense, the Subject acts as a driving force behind political movements, revitalizing political consciousness. There is thus a correlation between the Subject and the movements: both organize their actions with the aim of altering situations they no longer tolerate or endorse.

In this regard, it is worth considering the three elements Michel Wieviorka employs in the “triangle of differences,” as the essential components of diversity and the possible configurations of differences, based on the combination of the triangle’s angles, correspond to social elements (Wieviorka and Ohana, 2001).

The first vertex represents collective identity, understood as the cultural references that foster a sense of belonging to a group or community (Polletta and Jasper, 2001; Crespi, 2015). The traits within collective identities serve as guidelines for social action. The second vertex pertains to individualism, which positions each person as an elementary atom within a society where individuals, theoretically free and equal before the law, engage as separate beings in modern life. The final component of the triangle refers to the Subject, who seeks to find their place in society by expressing their creativity, humanity, sensitivity, and uniqueness (Wieviorka, 2005b).

When considering Wieviorka’s triangle of differences, it becomes evident that the three elements he identifies in analyzing social differences closely resemble the elements of working-class consciousness (Touraine, 1965) and social movements (Melucci, 1989, 1995; Farro, 1998), but they can also be applied to the subject.

**Table 1:** 

Principles	Worker consciousness	Social movements	Triangle of differences
Identity principle	Worker considers himself the producer and provider of an essential factor of production	Defense of the identity and interests of the movement	The subject
Principle of opposition	Worker identifies the adversary, highlighting the obstacles that prevent him from appropriating his works	He fights against an opponent who is generally an expression of consolidated politics and which he wants to change	Individualism, which makes each person the elementary atom of a society in which men participate as many individual beings
Principle of totality	The field of social conflicts is defined	Common vision with which the movement is identified	The set of cultural references on which the feeling of belonging to a group or community is based and which becomes an orientation for social action

The Subject, therefore, commits to promoting and sharing an open and fluid collective identity that unites different components and aligns with emerging global movements seeking representation (Daher, 2013). This collective identity enables the Subject to act and construct its existence consciously and responsibly, moving away from exclusive and totalizing identities that lead to individualism, which it must oppose. A cultural and creative agent seeking to take ownership of its own destiny, demonstrating the capacity, and the willingness, to transform its reality starting from within, while simultaneously engaging with others and valuing a *community* with it identifies. This social actor, understood as a “Subject,” can thus attempt to challenge social dynamics within a context where less recognizable and more insidious forms of domination emerge, potentially leading to a process of de-subjectification. To counter a socio-cultural drift that imposes standardized, homogenizing roles thereby hindering the full realization of subjectivity (Touraine, 2015). It is necessary to move beyond a state of vacuity characterized by transience and contingency, and instead pursue a genuine, substantial path toward empowerment and self-assertion (Mongardini, 2009). By revisiting the biographical elements of the Subject’s identity, it becomes possible to resume collective action; group members, as well as members of social and political movements, bound by shared interests and mutual solidarity, can then define themselves and consciously, responsibly give meaning to their existence and their unified actions.

## Discussion

The changes currently ongoing are undoubtedly more challenging than those that accompanied the transition between stages of modern industrial society; they increasingly distance us from the utopia of a society characterized by the absence of risk, by abundance, progress, well-being, and perfection. Faced with the consequences of an irresponsible economic-financial model and the weakness of institutions, which seem no longer capable of effective planning, many feel the need to engage with a broad analytical perspective (Milanovic, 2019; Ricciardi, 2011). This need appears all the more urgent during a transitional phase in which prevailing models, fueled by individualism, have proven inadequate and unreliable, causing that once-unconditional faith in progress to fade. There is talk of a “third wave” of modernization, just as the “end of the social” is hypothesized (Touraine, 2013), prompting the legitimate question of what, or who, might replace society. In reality, the goal is not to find an alternative to society, but rather to shift the fulcrum toward something that is not excessively weak or precarious. For some time now, the importance of rediscovering the emotional and creative spheres has been gaining renewed appreciation; thus, a possible alternative to an increasingly uncertain society might lie in the “Subject,” an entity that shuts the door on individualism to open it to a dimension that is human, plural, collective, and, consequently, ontologically social. Indeed, the Subject does not serve causes, values, or laws other than its own need and desire to resist fragmentation within a shifting universe devoid of order and balance; instead, its primary objective is personal freedom and ontological security (Nussbaum, 2011). A cultural and creative agent that attempts at the very least to take ownership of its own destiny, demonstrating the ability to choose, decide, confront, and even transform negative situations by starting with itself, while necessarily engaging with others.

In this perspective, the defense of human rights must become a priority response, both individual and collective, aimed at restoring meaning to society. However; yet it can only be effective if it succeeds in ensuring the affirmation of three principles proposed by the “triangle of differences”: identity, recognition of opposition, and totality. Indeed, the principle of totality, associated with culture, together with the identity of belonging realized within the community, can identify the arena of social conflicts, thereby proposing a hypothetical solution characterized by the identification and participation of the Subject.
